# Interventions Integrating Mental Health Services Into HIV Care in Africa; a Scoping Review

**DOI:** 10.3389/ijph.2025.1608137

**Published:** 2025-07-07

**Authors:** Charlotte Nwogwugwu, Chinedum Favor, Yunting Fu, Theddeus Iheanacho

**Affiliations:** ^1^ School of Nursing, University of Maryland, Baltimore, MD, United States; ^2^ School of Public Health, University of New Haven, West Haven, CT, United States; ^3^ School of Public Health, Yale University, New Haven, CT, United States

**Keywords:** global health education, HIV, mental health and psychosocial support, behavioral and psychosocial problems, health promotion intervention

## Abstract

**Objectives:**

This scoping review aims to synthesize existing evidence on mental health (MH) interventions for people living with HIV/AIDS (PLWHA) in Africa. Given the high prevalence of MH disorders in this population and barriers to care, integrating MH services within HIV care settings is explored as a potential strategy to improve patient outcomes.

**Methods:**

Following PRISMA-ScR guidelines, a systematic search was conducted across five databases to identify studies examining MH interventions for PLWHA in African settings. Studies meeting inclusion criteria were reviewed for intervention type, target population, and reported outcomes.

**Results:**

Of 818 studies identified, 16 from six African countries met inclusion criteria, with Zimbabwe and South Africa leading in interventions. Most targeted depression, employing non-pharmacological approaches such as task-sharing and stepped-care models. Findings suggest integrated MH and HIV care improves MH symptoms and adherence to antiretroviral therapy.

**Conclusion:**

Despite limited studies, evidence supports the feasibility and benefits of integrating MH services into HIV care in Africa. Scaling evidence-based interventions is essential to address unmet MH needs in this population.

## Introduction

The prevalence of mental, neurological, and substance use (MNS) disorders is on the rise globally with approximately 450 million people suffering from mental and behavioral disorders, and approximately one in four persons developing such a disorder during their lifetime [1, 2]. It is estimated that between 1990 and 2019 the Disability Adjusted Life Years (DALY) increased from 80.8 million to 125.3 million globally. More than 80% of people with MNS disorders live in Low and middle income countries (LMICs) [1]. However healthcare systems in LMICs possess far less capacity to address such conditions than High Income Countries (HICs) [3]. For instance, Nigeria, Africa’s most populous nation and an LMIC [4], has over 7 million people with depression and over 4 million people with anxiety disorders, the highest number of cases compared to other countries in the African region [5].

Although data on the prevalence of mental health conditions in Northern African countries is limited, available data that combines statistics from both Middle Eastern and Northern African countries indicates a significant increase of approximately 35% in the prevalence of mental health conditions, particularly anxiety and mood disorders, between 1990 and 2019 and this increase has been more pronounced among females [6]. In Southern Africa, particularly in South Africa where the prevalence of depression is reported at 25.7% and as high as 38.8% in the Northern Cape area, the highest rates are observed among women and those aged 65 years and older [7]. In Eastern African countries like Rwanda, it is estimated that approximately 20% of the population live with at least one mental health condition, with depression being the most prevalent, affecting 23% of women compared to 16% of men [2].

On the African continent overall, the point, period, and lifetime prevalence rates for major depressive disorders ranged from 2.0% to 33.2% for current diagnoses, 1.1%–7.1% for diagnoses within the past year, and 0.3%–26.2% for lifetime prevalence. [8]. These data points are worse among people living with HIV/AIDS (PLWHA) in Africa. They are three times more likely than the general population to have mental disorders [7]. For example depression, the most common mental disorder occurring among PLWHA, has a prevalence ranging from 9% to 32% in sub-Saharan Africa [7]. These prevalence rates are most likely under reported due to stigma associated with seeking care and the lack of available and affordable services.

It is estimated that approximately 50% of PLWHA in Africa meet diagnostic criteria for mental and substance use disorders though these remain under-diagnosed and under-treated [7]. Substance use disorder research has received limited attention in African countries. In a study from 12 African nations, it is estimated that the lifetime prevalence of alcohol use disorder ranges from 1.2% to 14%, while drug use disorder is approximately 1.0%–4.5% [7]. These prevalence rates are also most likely under reported due to stigma associated with seeking care and the lack of available and affordable services [7]. Current estimates show that among PLWHA, in Africa the prevalence rate of suicide is 2.3% for suicide attempts and 2.9% for suicide ideation [9].

The bidirectional interaction between mental, neurological, and substance use (MNS) conditions and HIV is well-documented. Certain MNS conditions, such as depression and substance use disorders, may increase vulnerability to HIV due to factors like impaired judgment and risky behaviors. Conversely, people living with HIV (PLHIV) face a higher risk of developing mental health conditions due to biological, psychological, and social stressors associated with the illness [10]. Individuals with mental, neurological, and substance use (MNS) conditions face an increased likelihood of experiencing poverty and sexual violence, engaging in transactional sex, and practicing inconsistent condom use [11]. These vulnerabilities are exacerbated by structural barriers such as limited access to mental healthcare, economic instability, systemic gender inequalities, stigma, and inadequate social support systems [11]. Additionally, legal and policy gaps, discrimination within healthcare settings, and the criminalization of survival strategies, such as sex work, further entrench these risks. Addressing these structural determinants through policy reform, economic empowerment programs, and integrated mental health and sexual health services is crucial to reducing disparities and improving health outcomes among individuals with MNS conditions [12]. In addition, adherence to HIV treatment is significantly undermined by mental health conditions leading to, antiretroviral resistance and increased risk of mortality [12, 13]. Furthermore, some side effects from antiretroviral medications can also lead to mental health conditions thus, making PLWHA highly vulnerable to mental illness. Documented side effects from antiretroviral medications include but is not limited to psychosis, mood instability and mania, [10].

Despite the strong evidence linking HIV infection and AIDS to mental disorders there is a major lack of access to mental healthcare services for PLWHA in Africa with very minimal integration of these services in HIV care. This has negative consequences like delayed initiation of antiretroviral treatment (ART), poor ART adherence [14, 15], poor retention in care [16], accelerated disease progression [17], low rates of viral suppression and untimely death due to complications of this chronic but treatable condition. With these effects on the HIV cascade, untreated mental disorders are a critical but potentially modifiable determinant of optimal HIV treatment outcomes [18, 19].

Historically, in most African countries, mental health services have been provided in stand-alone psychiatric hospitals or outpatient clinics; usually in large urban centers [20]. These services are often expensive and inaccessible to most of the population despite mental healthcare access being a major component of the World Health Organization (WHO)’s Sustainable Development Goals [20]. There is evidence that targeted and integrated mental health interventions are feasible, and effective for PLWHA. In a meta-analysis of 29 studies conducted by Sin, N., and Dimatteo, R. (2014) there was evidence that implementation of robust mental health interventions and depression treatment for PLWHA led to significantly higher adherence rate to antiretroviral regimens [21].

Furthermore, evidence shows that among patients receiving interventions focused on alleviating the symptoms of depression, there was a stronger association with improved mental health outcomes compared to interventions with mental health as a secondary concern [22]. Other healthcare models such as collaborative, stepped-care which adopts a task sharing model by providing support to non-medical personnel in making appropriate decisions on the initiation of antidepressants, dose, duration and switching demonstrated significant promise in improving health outcomes among participants [23]. Outcomes showed an improvement in CD4 counts and adherence and remission of depressive symptoms at four-month follow-up [23].

This scoping review synthesizes and describes studies of mental health interventions for PLWHA in Africa published from 2010 to 2023.

## Methods

### Approach

The approach involved a 5-stage process: identifying the research question; identifying relevant studies; selecting studies; charting the data; collating, summarizing and reporting the relevant results.

### Search Strategy

Before the initial search, authors thoroughly went through a list of potential terms for the concept of HIV/AIDS, mental health interventions and geography areas to be included. After the discussion and testing, the search was conducted by the medical librarian using PubMed via NLM, Embase via Elsevier, CINAHL via EBSCOhost, APA PsycInfo via EBSCOhost, and Scopus to perform the literature search. The search queries were developed and tailored to each database incorporating keywords and indexed terms considered by our team to describe the f above mentioned concepts (see appendix). Considering the purpose of the scoping review, the search did not limit any publication period. The final search was run and completed on 28 February 2023 of the 5 databases resulting in 1010 records which narrowed down to 821 records after removal of duplications by Covidence^©^. We also reviewed bibliographies from included studies. This study was reported according to the Preferred Reporting Items for Systematic Reviews and Meta-Analyses extension for Scoping Reviews (PRISMA-ScR) [24] We did not include grey literature in the review due to feasibility reasons such as difficulty in identification, acquisition, processing and accessing these grey literature than the conventional literature. Furthermore, in line with expert recommendations on scoping reviews, we limited our search to peer-reviewed articles because they provide stronger scientific sources, which would inform more acceptable evidence-based recommendations for the academic community [25]. Each co-author independently reviewed the manuscripts. In cases where there were discrepancies regarding the acceptance or rejection of a manuscript, we discussed our evaluations to identify its strengths and limitations. A total of 31 studies underwent full-text review, of which 15 were subsequently excluded, resulting in 16 final articles included in this scoping review. Table 1 provides a comprehensive list of the included studies along with brief summaries.

**TABLE 1:** African studies included in scoping review (Ethiopia, Malawi, Nigeria, South Africa, Uganda, Zimbabwe. 2024).

Author(s)	Year of study	Who administered the intervention	Description of intervention	Age	Sex	Mental health condition	Country/Region of study
Duffy, Sharer, Cornman, Pearson, Pitorak and Fullem	2017	Nurses, community health workers, and traditional medicine practitioners	Stepped-care mental health/HIV integration	Not specified	Not specified	Depression, harmful alcohol, and substance use	Zimbabwe
Magidson, Joska, Belus, Andersen, Regenauer, Rose, Myers, Majokweni, Cleirigh and Safren	2021	Peers	Khanya, a task-shared, peer-delivered behavioral intervention for ART adherence and AOD reduction	Not specified	Not specified	Alcohol and other drug use (AOD)	South Africa
Chibanda, Shetty, Tshimanga, Woelk, Stranix-Chibanda and Rusakaniko	2014	Trained peer counselors	Group problem-solving therapy (PST) and amitriptyline	Postpartum mothers (average age not specified)	Female	Postnatal depression (PND)	Zimbabwe
Udedi, Stockton, Kulisewa, Hosseinipour, Gaynes, Mphonda, Mwagomba, Mazenga, Pence	2018	clinic staff with task-shifting approach	Integrating depression screening and treatment into HIV care initiation	Not specified	Not specified	Depression in people initiating ART	Malawi
Magidson, Andersen, Satinsky, Myers, Kagee, Anvari, Joska	2020	HIV care providers and substance use treatment therapists	Adaptation of Behavioral Activation (BA) therapy for substance use in HIV care	Not specified	Not specified	Substance use and its impact on HIV treatment adherence	South Africa
Madhombiro, Kidd, Dube, Dube, Mutsvuke, Muronzie, Zhou, Derveeuw, Chibanda, Chingono, Rusakaniko, Hutson, Morse, Abas, Seedat	2020	General nurses from the clinics	8–10 sessions of Motivational Interviewing blended with brief Cognitive Behavioral Therapy (MI-CBT) vs 4 Enhanced Usual Care (EUC) sessions	Mean age 43.3 years (SD = 9.1)	78.6% male (n = 184)	Alcohol Use Disorders (AUD) in people living with HIV (PLWH)	Zimbabwe
Nakimuli-Mpungu, Wamala, Okello, Alderman, Odokonyero, Mojtabai, Mills, Kanters, Nachega, Musisi	2015	trained facilitators	Group support psychotherapy (GSP) vs group HIV education (GHE); 8 weekly sessions	19 years or older	Not specified	Major depression in people with HIV	Uganda
Chibanda, Mesu, Kajawu, Cowan, Araya, Abas	2011	Trained female lay workers	Friendship Bench Intervention: 6 sessions of problem-solving therapy (PST) with activity scheduling	Adults (exact age range not specified)	Not specified	Common Mental Disorders (CMD)	Zimbabwe
Stranix-Chibanda, Chibanda, Chingono, Montgomery, Wells, Maldonado, Chipato, Shetty	2005	Not specified	Screening for psychological morbidity using the 14-item Shona Symptom Questionnaire (SSQ) at initial antenatal care visit	Pregnant women (exact age range not specified)	Female	Psychological morbidity in HIV-infected and uninfected pregnant women	Zimbabwe
Parcesepe, Mugglin, Nalugoda, Bernard, Yunihastuti, Althoff, Jaquet, Haas, Duda, Wester, Nash	2018	HIV clinic staff at surveyed sites	Screening and management of mental health and substance use disorders (MSDs) integrated into HIV care	Not specified	Not specified	Depression, PTSD, SUDs, and other mental health disorders in people living with HIV/AIDS (PLWHA)	sub-Saharan Africa
Iheanacho, Obiefune, Ezeanolue, Ogedegbe, Nwanyanwu, Ehiri, Ohaeri, Ezeanolue	2015	Trained church-based health advisors	Mental health screening using the 12-item General Health Questionnaire integrated into a community-based program for prevention of mother-to-child transmission of HIV	Pregnant women and their male partners (exact age range not specified)	Both (specific numbers not provided)	Psychological distress	Nigeria
Ahmed, Weldegebrea and Mekonnen	2020	Trained lay healthcare workers and clinicians	Integration of mental health services into HIV care using task-sharing: screening by lay healthcare workers and referral to clinicians for diagnosis and treatment	Not specified	Not specified	Mental health disorders in people living with HIV (PLHIV)	Ethiopia
Passchier, Owens, Wickremsinh, Bismilla, Ebuenyi	2019	Ten experts and local stakeholders from the UK and South Africa	Digital depression screening using the Mood in Retroviral Positive Individuals Application Monitoring (MIR + IAM) tool embedded in HIV primary care settings	Not specified	Not specified	Major depressive disorder in people living with HIV.	South Africa
Van Deventer	2015	Researchers conducting empowerment evaluation in the North West province, South Africa	Empowerment evaluation to engage patients in integrating and improving services for chronically ill patients (NCDs, HIV, mental illnesses) at primary healthcare (PHC) clinics	Not specified	Not specified	Non-communicable diseases (NCDs), HIV, and mental illnesses	South Africa
Okello, Ngo, Ryan, Musisi, Akena, Nakasujja, Wagner	2012	Researchers conducting in-depth interviews with 26 adult HIV-positive clients receiving ART in Uganda	In-depth interviews using a semi-structured interview guide to explore how HIV-positive individuals conceptualize and describe depression, its manifestation over time, and the impacts of ART and antidepressant treatment	Adult HIV-positive clients (specific ages not provided)	Not specified	Depression among HIV-positive individuals	Uganda
Everitt-Penhale, Kagee, Magidson, Joska, Safren, Cleirigh, Witten, Lee, Andersen	2019	Nurses in two peri-urban HIV clinics near Cape Town, South Africa	Adapted cognitive-behavioural therapy (CBT) treatment for adherence and depression, task-shifted to nurses	Not specified	isiXhosa-speaking females	Depression and adherence to ART among HIV patients	South Africa

### Inclusion Criteria

Studies were included only if a mental health intervention (Screening, assessment, treatment or referral) among people living with HIV on the African continent was provided.

### Exclusion Criteria

Studies conducted outside of the African continent were excluded. Studies on HIV prevention and studies which included people who are HIV negative were excluded.

### Study Selection

A total of 818 manuscript abstracts were reviewed independently by the authors to prevent selection bias and to determine if they met the criteria for inclusion. Only 28 manuscripts matched between the independent reviews. We then reviewed the 28 manuscript more thoroughly and eliminated papers that were only abstracts, and those that did not include any mental health services for people living with HIV.

### Data Synthesis

We charted the evidence by utilizing a data extraction table. This approach allowed us to extract author names, year, article title, country, study design, population studied, and interventions provided. We utilized maps which depicted the countries where the studies were conducted and tables to summarize the characteristics of the studies.

## Results

### Search Results

**FIGURE 1:**
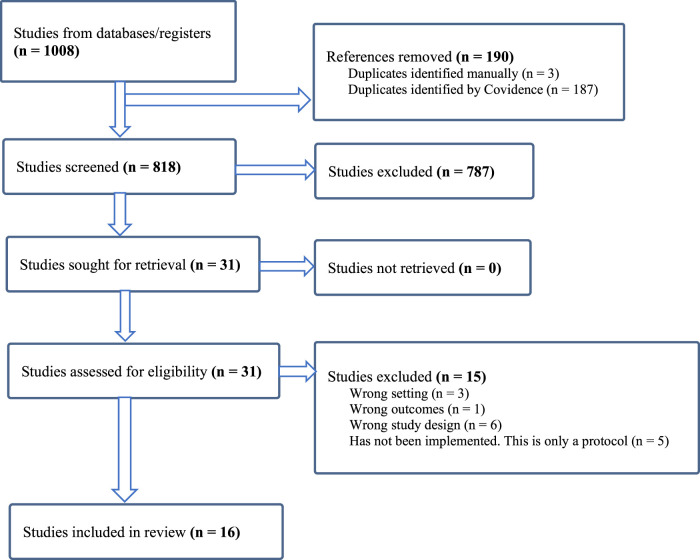
Search Strategy and inclusion and exclusion of studies (Ethiopia, Malawi, Nigeria, South Africa, Uganda, Zimbabwe. 2024).

Of the 818 studies initially identified, 787 were excluded for being irrelevant to study questions and additional 16 were excluded for not meeting inclusion criteria. A total of 16 studies from 6 countries were included (Figure 1).

### Characteristics of Included Studies

**FIGURE 2:**
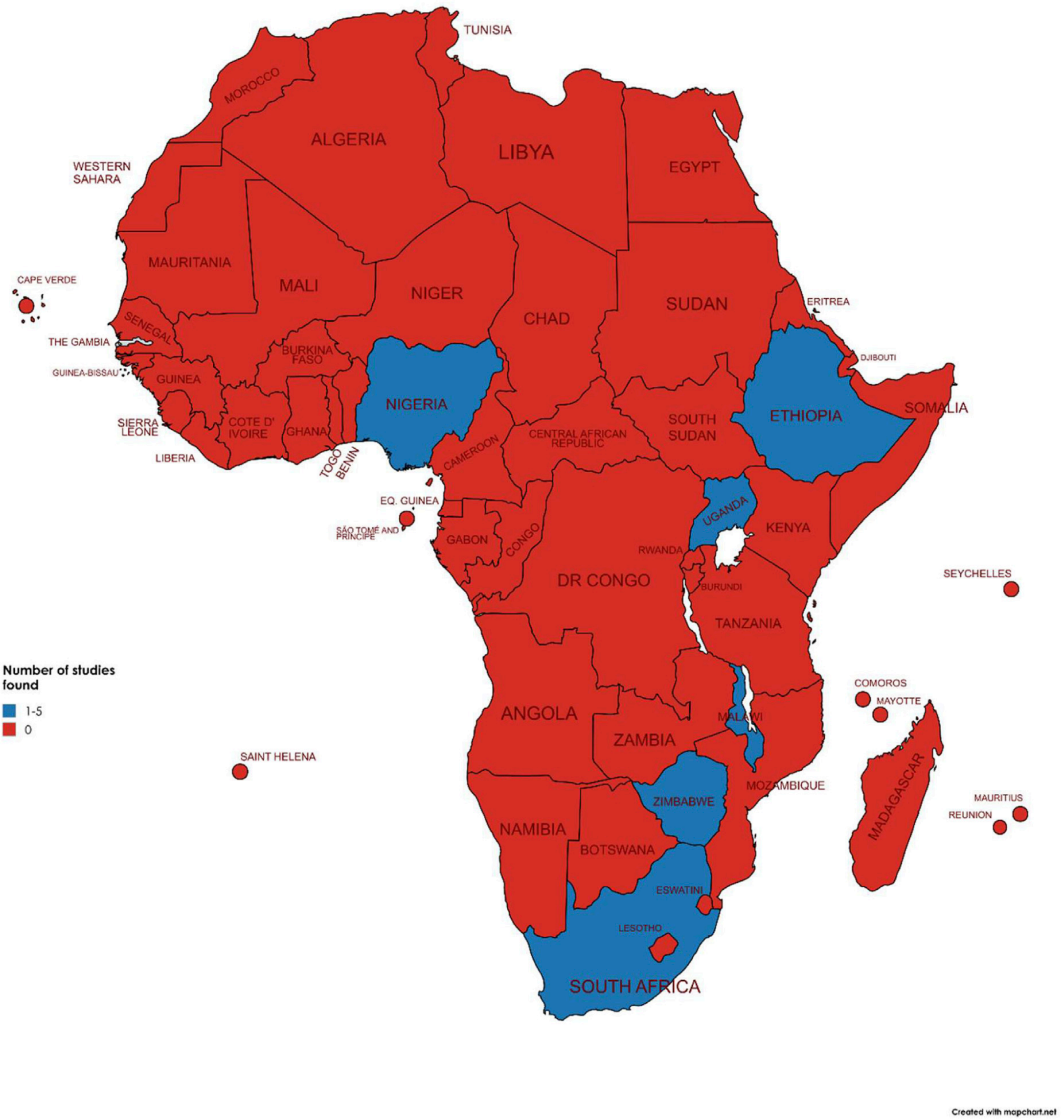
Map of Africa (Ethiopia, Malawi, Nigeria, South Africa, Uganda, Zimbabwe. 2024).

The included studies originated mainly from 6 countries (See Figure 2), with Zimbabwe (5/16, 31%) and South Africa (5/16, 31%) having a tie for the highest number of published mental health interventions, followed by Uganda (2/16, 13%), then Ethiopia (1/16, 6%), Malawi (1/16, 6%), and Nigeria (1/16, 6%), One study had examined mental health intervention in 29 LMICs that included sub-Saharan countries (1/16, 6%). The most common mental health condition addressed within the studies was depression (12/16, 80%) with the rest of the studies focusing on substance use disorders (3/16, 20%). Majority of the studies were of non-pharmacological interventions (14/16, 94%). Only one study had a pharmacological component, however this was utilized only when the patient was referred to a prescriber for specialist care and prescribing of antidepressants (1/16, 6%). Table 1 illustrates the description of all studies included in the review.

## Discussion

The studies reviewed demonstrate a consistent effort to integrate mental health interventions into HIV care across various settings in Africa. These interventions often utilized task-shifting approaches, where responsibilities typically held by specialized healthcare providers were shared with trained lay workers, peers, or community health workers collaboratively in stepped-care approach. This strategy was effective in broadening access to mental health services, particularly in resource-limited settings [26, 27]. For instance, the Friendship Bench intervention in Zimbabwe and the group support psychotherapy (GSP) in Uganda showed significant reductions in symptoms of common mental disorders and major depression, respectively [23, 26], highlighting the potential of these community-based approaches in addressing mental health needs.

The studies also explored innovative methods for delivering mental healthcare, such as the integration of digital tools and behavioral therapies within HIV care settings. The Khanya intervention in South Africa, a peer-delivered behavioral program, demonstrated promising outcomes in improving ART adherence and reducing substance use [22]. Similarly, the digital depression screening tool embedded in HIV primary care in South Africa effectively identified major depressive disorder among people living with HIV [12], suggesting that technology can play a crucial role in mental health screening and intervention, especially in regions where healthcare resources are scarce.

Overall, these interventions underscore the feasibility and effectiveness of integrating mental health services with HIV care, particularly through task-sharing and the use of existing community-based resources [28]. While the specific outcomes and effectiveness metrics varied across studies, the general trend indicated positive impacts on mental health conditions and adherence to HIV treatment particularly increasing access to screening opportunities which increased the likelihood of getting treatment. The success of these interventions in diverse contexts suggests that such models could be scalable and adaptable to other regions with similar healthcare challenges, providing a comprehensive approach to managing the dual burden of HIV and mental health disorders [11, 29].

The interventions addressing behavioral and psychological concerns among people living with HIV (PLWH) in Africa are notably diverse, reflecting the varied socio-economic and cultural contexts within which they are implemented. This diversity spans the types of interventions, the methods of delivery, the personnel involved, and the specific mental health conditions targeted.

The integration of mental health services into HIV care is a common strategy. This integration often leverages existing healthcare structures and resources, utilizing task-sharing approaches where non-specialist health workers, such as nurses, community health workers, and even peer counselors, are trained to deliver mental health interventions. For instance, the *Friendship Bench Intervention* in Zimbabwe [26], is a notable example where trained female lay workers provided problem-solving therapy (PST) and activity scheduling to individuals suffering from mild to moderate mental disorders. This intervention was implemented in primary healthcare centers, ensuring that mental health services were accessible to a broader population within the community.

The interventions also varied significantly in terms of their therapeutic approaches. Some interventions, such as the Khanya program in South Africa, focused on behavioral interventions that are peer-delivered, targeting both ART adherence and the reduction of alcohol and other drug use [22]. This program demonstrates the effectiveness of peer involvement in mental healthcare, especially in settings where traditional mental health services might be underdeveloped or overstretched.

Other interventions were more structured and formalized, such as the Motivational Interviewing blended with Cognitive Behavioral Therapy (MI-CBT) used in Zimbabwe. This intervention, administered by general nurses, was designed to address Alcohol Use Disorders (AUD) in PLWH. The program involved multiple sessions and was implemented in clinic clusters, which highlights the adaptability of such interventions in different healthcare settings [29].

The diversity of these interventions is also reflected in the mental health conditions they target, ranging from depression, addressed in the *Group Support Psychotherapy* (GSP) intervention in Uganda [23], to alcohol and substance use disorders, as addressed by the Behavioral Activation (BA) therapy adaptation in South Africa [30]. The variability in these interventions and their delivery mechanisms underscores the complexity of mental health issues among PLWH in Africa, necessitating tailored approaches that consider the specific needs and contexts of different populations.

The studies on mental health interventions for PLWHA were concentrated in few African countries. This can be attributed to several key factors, including disparities in research infrastructure, governmental prioritization, and international collaborations. Countries like South Africa, Zimbabwe, and Uganda have emerged as leaders in this field primarily due to their relatively advanced research infrastructures. South Africa, for instance, has a robust network of universities, research institutions, and healthcare facilities that are well-equipped to conduct large-scale studies compared to other African countries. The country’s government has also prioritized mental health through policies such as the National Mental Health Policy Framework and Strategic Plan (2013–2020), which has created a conducive environment for research and intervention development.

Furthermore, international partnerships play a significant role in the geographical concentration of research. South Africa, for example, has established numerous collaborations with international research bodies and non-governmental organizations (NGOs), which often provide the funding and technical expertise necessary for such studies. This is evident in the numerous studies conducted in South Africa that focus on integrating mental health into HIV care settings, such as the adaptation of cognitive-behavioral therapy (CBT) for ART adherence and depression [9].

In contrast, countries with less developed research infrastructure, limited funding, and fewer international partnerships may struggle to produce and publish research at the same rate. This results in an uneven distribution of studies across the continent. For instance, despite the significant HIV burden in countries like Nigeria and Ethiopia, research output on the mental health interventions for PLWHA in these countries is relatively lower. This disparity underscores the importance of investment in research capacity building in less represented countries to ensure a more balanced understanding of mental health issues for PLWHA across the continent [14, 31].

Governmental investments in mental health services in Africa vary significantly across countries, reflecting differences in economic capacity, policy priorities, and health system infrastructure. In countries where mental health has been prioritized, there has been notable progress in integrating these services into broader healthcare systems, particularly in the context of HIV care [32].

Zimbabwe serves as a key example where governmental and non-governmental collaborations have resulted in innovative mental health interventions. The *Friendship Bench* program, which started in Harare, has been scaled up to reach over 60,000 people with over 20 districts and over 200 primary healthcare facilities due to its success in treating common mental disorders at the primary care level. This initiative has received support from the government, which has been instrumental in its expansion to other regions of the country, demonstrating a commitment to addressing mental health needs among its population [26].

In South Africa, the government’s investment in mental health services has been more structured, particularly through the integration of mental health into HIV care. The National Mental Health Policy Framework and Strategic Plan (2013–2020) laid the groundwork for substantial improvements in mental health services, including the development of guidelines for the integration of mental health into primary care settings. The South African government’s efforts have been supported by international partners, further boosting the country’s capacity to implement and scale mental health interventions within the HIV care framework [33].

However, in many African countries, governmental investment in mental health remains minimal, often limited by competing public health priorities and constrained budgets. In these contexts, mental health services are frequently supported by international donors and non-governmental organizations (NGOs), with governments playing a more limited role in funding and implementation. This reliance on external funding can lead to sustainability issues, particularly when donor priorities shift, or funding diminishes [34].

Significant gaps persist in mental health services for people living with HIV (PLWH) in Africa, particularly concerning the integration and accessibility of care delivery. For instance, ongoing challenges remain in addressing substance use disorders among PLWH in South Africa, despite the implementation of Behavioral Activation (BA) therapy within HIV care settings [30]. The study underscores that while adaptations of mental health interventions have been made to fit the local context, issues such as stigma, limited access to trained mental health professionals, and the complexity of co-occurring disorders continue to hinder the effectiveness of these interventions.

Similarly, interventions like Motivational Interviewing blended with Cognitive Behavioral Therapy (MI-CBT) in Zimbabwe has shown promise in the continuity of care but is often disrupted by factors such as resource constraints, the stigma associated with mental health disorders, and the geographical inaccessibility of services for those living in rural or peri-urban areas [29]. This highlights a critical gap in the mental healthcare of PLWH, where services are either unavailable or inadequately delivered to those most in need. The combined results of these studies indicate that, despite some progress in incorporating mental health services into HIV care in Africa, significant obstacles remain. These obstacles include the need for enhanced, expanded and sustained interventions, better access to mental health services, and stronger initiatives to reduce the stigma associated with mental health issues among PLWH. Overcoming these obstacles is critical for enhancing the overall mental health and wellbeing of this vulnerable group.

A limitation of this scoping review is that, while we have summarized the findings and intervention outcomes of the studies included, we did not re-analyze the aggregated results of the interventions. Assessing evidence, quality or study biases is not typically required in a scoping review. Nevertheless, the findings have highlighted significant gaps in the existing literature.

A key strength of this review is the thorough search process, which was conducted in collaboration with a professional librarian YF and involved the identification of relevant studies across multiple, robust databases.

### Conclusion

This scoping review analyzed peer-reviewed literature on mental health interventions for people living with HIV (PLWHA) in Africa, aiming to assess the availability, effectiveness, and implementation of such interventions. The findings reveal a significant gap in research, with only a limited number of studies addressing mental health support for this population. Given the high burden of mental health disorders among PLWHA, including depression and anxiety, there is a critical need for the development and rigorous evaluation of culturally relevant, evidence-based interventions. Additionally, existing interventions that have demonstrated effectiveness should be scaled up to enhance accessibility and sustainability within the African context. Strengthening mental health services for PLWHA through integrated healthcare approaches, policy support, and community-based strategies is essential to improving overall wellbeing and treatment outcomes. There is a critical need for further research on the influence of cultural factors on mental health outcomes, particularly in low- and middle-income countries, where existing studies remain limited. Culture plays a fundamental role in shaping individuals' perceptions of mental health, their willingness to seek care, and the effectiveness of various interventions. However, the lack of robust data on these relationships hinders the development of culturally tailored mental health strategies. By generating strong, evidence-based research that examines how cultural norms, beliefs, and practices affect mental health outcomes, we can significantly enhance existing interventions. This research presents an opportunity to develop more inclusive, context-specific mental health programs that resonate with diverse populations. Furthermore, by ensuring that mental health policies and interventions are culturally responsive, we can improve accessibility, reduce stigma, and promote better mental health outcomes globally. Addressing this gap in knowledge will not only advance scientific understanding but also foster more equitable and sustainable mental healthcare solutions worldwide.
